# Comprehensive investigations of 2-phenylethanol production by the filamentous fungus *Annulohypoxylon stygium*

**DOI:** 10.1007/s00253-024-13226-y

**Published:** 2024-06-15

**Authors:** Qianwen Tong, Lizhi Yang, Jinxiang Zhang, Yue Zhang, Yuji Jiang, Xinrui Liu, Youjin Deng

**Affiliations:** 1 Mycological Research Center, College of Life Science, Fujian Agriculture and Forestry University, Fuzhou, 350002 China; 2 College of Food Science, Fujian Agriculture and Forestry University, Fuzhou, 350002 China

**Keywords:** *Annulohypoxylon stygium*, 2-phenylethanol, L-phenylalanine, Ehrlich pathway

## Abstract

**Abstract:**

2-Phenylethanol (2-PE) is an aromatic compound with a rose-like fragrance that is widely used in food and other industries. Yeasts have been implicated in the biosynthesis of 2-PE; however, few studies have reported the involvement of filamentous fungi. In this study, 2-PE was detected in *Annulohypoxylon stygium* mycelia grown in both potato dextrose broth (PDB) and sawdust medium. Among the 27 *A. stygium* strains investigated in this study, the strain “Jinjiling” (strain S20) showed the highest production of 2-PE. Under optimal culture conditions, the concentration of 2-PE was 2.33 g/L. Each of the key genes in *Saccharomyces cerevisiae* shikimate and Ehrlich pathways was found to have homologous genes in *A. stygium*. Upon the addition of L-phenylalanine to the medium, there was an upregulation of all key genes in the Ehrlich pathway of *A. stygium*, which was consistent with that of *S. cerevisiae*. *A. stygium* as an associated fungus provides nutrition for the growth of *Tremella fuciformis* and most spent composts of *T. fuciformis* contain pure *A. stygium* mycelium. Our study on the high-efficiency biosynthesis of 2-PE in *A. stygium* offers a sustainable solution by utilizing the spent compost of *T. fuciformis* and provides an alternative option for the production of natural 2-PE.

**Key points:**

• *Annulohypoxylon stygium can produce high concentration of 2-phenylethanol.*

• *The pathways of 2-PE biosynthesis in Annulohypoxylon stygium were analyzed.*

• *Spent compost of Tremella fuciformis is a potential source for 2-phenylethanol.*

## Introduction

2-Phenylethanol (2-PE) is an aromatic alcohol with a rose-like fragrance (Qian et al. 2019). It has been used as a fragrance ingredient in various products, including food, perfumes, and plant preservatives (Chreptowicz et al. 2016; Mo and Sung 2007; Scognamiglio et al. 2012). The majority of 2-PE used in the industry is obtained by chemical synthesis. However, chemical synthesis is limited by toxic compounds and byproducts that are difficult to remove (Hua and Xu 2011; Martínez-Avila et al. 2020). Thus, the microbial biosynthesis process has received considerable attention as an alternative for 2-PE production and is a simpler and more efficient option for purification (Carroll et al. 2016).

The most efficient microorganisms for the production of 2-PE are yeasts, including *Saccharomyces cerevisiae* (Kim et al. 2014), *Kluyveromyces marxianus* (Lu et al. 2016), *Kluyveromyces lactis* (Qian et al. 2019), and *Pichia fermentans* (Chreptowicz et al. 2018). Yeasts are known to biosynthesize 2-PE via the shikimate or Ehrlich pathways (Wang et al. 2019). The shikimate pathway is a long pathway with multiple branches and various inhibitory feedback mechanisms and results in a low production of 2-PE (Hassing et al. 2019). However, when L-phenylalanine (L-Phe) is rich or serves as the sole nitrogen source, the Ehrlich pathway plays a leading role and produces higher output of 2-PE. The Ehrlich pathway consists of three steps: L-Phe is transformed into phenylpyruvate by a transaminase, then decarboxylated to phenylacetaldehyde by a decarboxylase, and finally reduced to 2-PE by a dehydrogenase (Dickinson et al. 2003). In *S. cerevisiae*, several genes are involved in the transformation of L-Phe into 2-PE via the Ehrlich pathway, including *ARO8*, *ARO10*, and *ADH*, which encode an aminotransferase, a decarboxylase, and a dehydrogenase, respectively (Dai et al. 2021; Hazelwood et al. 2008). Overexpression of *ARO8* and *ARO10* leads to an increase in the yield of 2-PE by 37% (Yin et al. 2015), and the co-expression of *ARO10* and *ADH* can significantly improve the yield of 2-PE by 6.5 folds (Shen et al. 2016). Few studies have reported the production of 2-PE by filamentous fungi.

*Annulohypoxylon stygium* is a white-rot filamentous fungus belonging to the family *Xylariaceae*. It exhibits a high ability to degrade lignin and carbohydrates (Hsieh et al. 2005; Wingfield et al. 2018). As an associated fungus, *A. stygium* provides nutrition for the growth and development environments of *Tremella fuciformis* and is commonly found in natural and artificial cultivation environments (Deng et al. 2016; Liu et al. 2019). More than 500 kt of fresh *T. fuciformis* were produced in 2020 (Sun 2023), generating more than 800 kt of spent mushroom compost, most of which contains only *A. stygium* mycelium, just less than 5% being a mixture of the two fungi. *A. stygium* has also been reported to be a good source of some active metabolites, such as melanin, oxidative stress resistance, and glycohydrolases (Liu et al. 2022; Robl et al. 2015; Wu et al. 2008).

In this study, we used *A. stygium* as material, detected 2-PE content in its volatile matter spectrum at different culture conditions, screened out the strain with most powerful biosynthesis of 2-PE from our culture collection, optimized medium composition and culture conditions, and analyzed the potential biosynthetic pathway of 2-PE production. Our purpose is to develop an alternative filamentous fungus for high-efficiency biosynthesis of 2-PE.

## Materials and methods

### Strains and media

The *T. fuciformis* Tr21 strain, a major cultivar contributing to most of the total production, as well as 27 *A. stygium* strains (Table 1) were obtained from the Center for Mushroom Germplasm Resources Management and Preservation of Fujian Province (CMMPF, Fujian, China). *A. stygium* S20 was also deposited in the China Center for Typical Culture Collection (CCTCC) with the accession number CCTCC NO:M 2020504 (Table 1). Potato dextrose broth (PDB) medium: 200 g/L potato infusion, 20 g/L glucose; Potato dextrose agar (PDA) medium: PDB medium plus 20 g/L agar. PDB + L-Phe medium: PDB medium plus 4 g/L L-Phe. Sawdust medium: 78% sawdust, 19.5% wheat bran, 1% sucrose, 1% gypsum, and 0.5% MgSO_4_.

**Table 1:** *A. stygium* strains used in this study

Strain code	Strain	Sources	Strain code	Strain	Sources
S1	Wuyi201502	CMMPF HP. sp 0001	S15	HPSP0043	CMMPF HP. sp 0015
S2	TJAS	CMMPF HP. sp 0002	S16	JZB2118076	CMMPF HP. sp 0016
S3	NL White	CMMPF HP. sp 0003	S17	TJY5	CMMPF HP. sp 0017
S4	NL Black	CMMPF HP. sp 0004	S18	HPSP0059	CMMPF HP. sp 0018
S5	HPSP0006	CMMPF HP. sp 0005	S19	Gutianjile	CMMPF HP. sp 0019
S6	T0052	CMMPF HP. sp 0006	S20	Jinjiling	CMMPF HP. sp 0020 CCTCC NO:M 2020504
S7	HPSP0004	CMMPF HP. sp 0007	S21	Xitouwei	CMMPF HP. sp 0021
S8	T0050	CMMPF HP. sp 0008	S22	Gulinyihao	CMMPF HP. sp 0022
S9	AS	CMMPF HP. sp 0009	S23	Wuyi2014	CMMPF HP. sp 0023
S10	Wuyi201501	CMMPF HP. sp 0010	S24	T0048	CMMPF HP. sp 0024
S11	T0054	CMMPF HP. sp 0011	S25	B21	CMMPF HP. sp 0025
S12	S1	CMMPF HP. sp 0012	S26	HPSP0032W	CMMPF HP. sp 0026
S13	AS Mingwei	CMMPF HP. sp 0013	S27	JZB2115093	CMMPF HP. sp 0027
S14	HPSP0032B	CMMPF HP. sp 0014			

### Odor chemical determination

As the associated fungus of *T. fuciformis* Tr21, *A. stygium* strain S2 was used to detect its odor chemicals in both PDB and sawdust medium. The mycelium was cultivated into the PDB medium for 4 days at 28 °C and 120 rpm, then harvested using the filtering paper method to obtain PDB sample. Sawdust sample was collected from bottom cultivation materials (including only *A. stygium* mycelia) after *T. fuciformis* cultivated in the sawdust medium at 23 °C for 34 days and the fruiting bodies being 6–8 cm in diameter. Both PDB and sawdust samples as well as their corresponding un-inoculated media were immediately used to determine their odor chemicals using a TOFMS/GC–MS (Agilent, Santa Clara, CA, USA).

### Screening of 2-PE-producing strains

Each of tested *A. stygium* strains was inoculated into 100 mL PDB + L-Phe medium and incubated in a constant temperature shaker at 28 °C at a shaking speed of 160 rpm for 6 days. The fermentation broth was centrifuged at 4000 rpm for 10 min, and the supernatant was filtered. The contents of L-Phe and 2-PE were determined using high-performance liquid chromatography LC–20AD (HPLC; Shimadzu, Kyoto, Japan) with a C-18 column (5 µm, 250 mm × 4.6 mm; Shimadzu, Shanghai, China). The mobile phase comprised 0.6% acetic acid aqueous solution and methanol, the flow rate was 0.7 mL/min, the temperature was set to 30 °C, and the detection wavelength was 258 nm.

All strains with 2-PE concentration more than 1.00 g/L were selected and evaluated their tolerance ability in the PDA medium. *A.stygium* mycelium was inoculated onto PDA medium containing 0 g/L (CK), 1 g/L, 2 g/L, and 3 g/L 2-PE, and incubated at 28 °C for 4 days. The strain with the highest tolerance was selected by measuring their mycelial growth rate.

### Morphological and ITS analyses

The mycelium of the S20 strain was cultured on PDA medium at 28 °C for 7 days. The mycelial morphology was examined using a bright-field microscope BX63 (Olympus, Tokyo, Japan). Primers ITS1 and ITS4 were used to amplify the ITS region of the S20 strain using a reaction system and operating conditions as described by Liu et al. (2022). The polymerase chain reaction (PCR) products were purified and sequenced by Sangon Biotech Co., Ltd. (Shanghai, China). The obtained ITS sequence (GenBank: PP140388) was used to search for the best hits of known species sequences for species identification using NCBI BLAST.

### Optimization of growth media for maximum production of 2-PE

The S20 strain was cultivated into PDB medium at 28 °C for 2 days. Optimization of 2-PE production was achieved in four steps, by evaluating the effects of different carbon sources, L-Phe consumption, and the concentration of potato infusions and MgSO_4_. Different carbon sources including glucose, maltose, lactose, mannitol, and sucrose were selected for 2-PE production. The S20 strain was incubated in PDB + L-Phe medium containing 20 g/L of the different carbon sources, 4 g/L of L-Phe, 200 g/L of potato infusion, and 0.1 g/L of MgSO_4_. The optimal carbon source concentrations ranged from 20 to 120 g/L. The L-Phe concentration ranged from 0 to 8 g/L, and the potato infusion content ranged from 200 to 1000 g/L. The MgSO_4_ concentrations ranged from 0 to 0.4 g/L. HPLC was used to determine 2-PE concentration in the fermentation broth.

Detailed optimization studies of 2-PE production were conducted using orthogonal experiments. Different concentrations of potato infusion, maltose, L-Phe, and MgSO_4_ were chosen as the factor levels (Table 2). Statistical analyses were carried out using IBM SPSS Statistics for Windows, Version 26.0 (IBM Corp., Armonk, N.Y., USA).

**Table 2:** Factor levels of the orthogonal test

Level	Variable			
	A Potato (g/L)	B Maltose (g/L)	C L-Phe (g/L)	D MgSO_4_ (g/L)
1	400	40	2	0.1
2	600	60	4	0.2
3	800	80	6	0.3

### Optimization of culture conditions

The S20 strain was grown for 2 days at 28 °C as described above on fermentation medium with different potato extraction times (5, 10, 15, 20, 25, 30 min), culture temperatures (22, 25, 28, 31, 34 °C), rotation speeds (120,140, 160, 180, 200 rpm), and liquid loading (60, 80, 100, 120, 140 mL); all the other components of the media remained the same. The optimal culture conditions were chosen based on the determination of 2- PE concentration by HPLC.

### RNA sequencing and data analyses

The S20 strain was grown in PDB and PDB + L-Phe medium at 25 °C, 150 rpm, for 4 days. Mycelium in both media was collected. Each treatment had three repetitions. Samples were grinded in a mortar with liquid nitrogen. RNA was extracted using the E.Z.N.A. Plant RNA Kit (Omega, Norcross, GA, USA) following the manufacturer’s instructions. RNA sequencing was performed by Novogene Corporation (Beijing, China) to obtain ~ 4 Gb clean data for each sample.

Clean sequencing reads were mapped on the *A. stygium* genome (GCA_003314315.1) using the STAR alignment software (Dobin et al. 2013). Differential gene expression analysis was performed using StringTie (Pertea et al. 2015) or Cufflinks (Trapnell et al. 2012) packages. Genes were considered to have statistically significant differential expression at *p* value < 0.05. The raw sequencing data were deposited in the National Center for Biotechnology Information (NCBI) Sequence Read Archive (SRA) with the BioProject accession number PRJNA1018449 (https://www.ncbi.nlm.nih.gov/sra/PRJNA1018449).

### Determination of biosynthetic pathway of 2-PE in *A. stygium*

Proteins (Ssy1p, accession number: NP_010444.1, ADH, accession number: NP_014032.1, ARO8, accession number: NP_011313.1 and PDR12, accession number: NP_015267.1) involved in the biosynthetic pathway of 2-PE in *S. cerevisiae* were downloaded from the NR database of the NCBI website. Local BLASTp was used to align these proteins against the proteomic database of *A. stygium* to determine their gene analogs. For each gene with multiple analogs, a phylogenetic tree was constructed to determine the closely related analogs using the program Mega 11 (Tamura et al. 2021) with the alignment option of Clustal-W and default parameters. Each candidate gene was further identified using RNA-seq analysis of the samples with or without additional L-Phe.

## Results

### Determination of odor chemicals

**Fig. 1:**
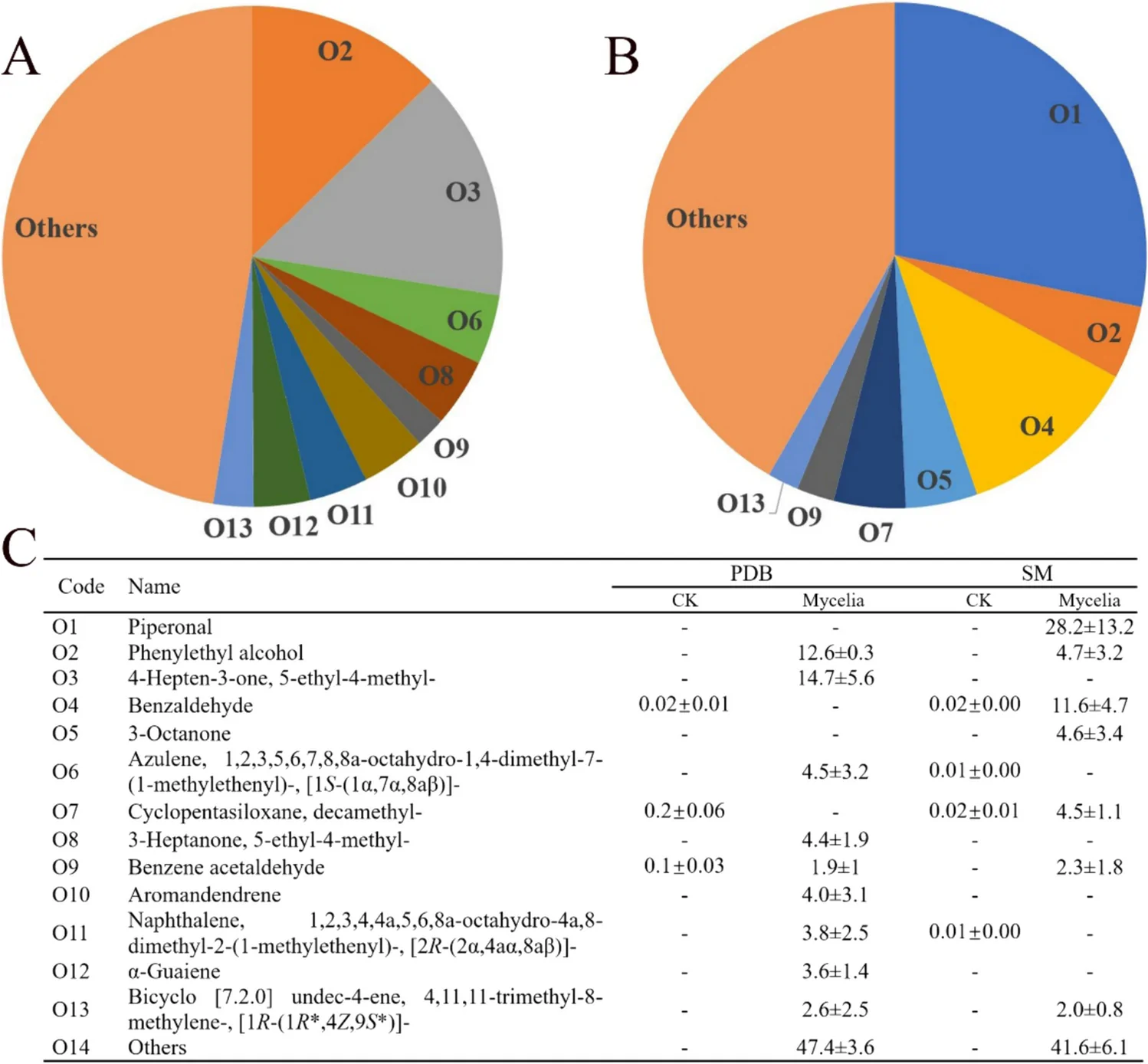
Odor chemicals produced by *A. stygium* (strain S2) in different media. **A, C** Proportion of odor chemicals emitted from samples in potato dextrose broth (PDB) medium after cultivated 4 days at 28 °C under 120 rpm. **B, C** Proportion of odor chemicals emitted from samples in sawdust medium (SM) when the fruiting bodies of *T. fuciformis* reaching 6–8 cm in diameter. CK, un-inoculated media

In an initial approach, the odor chemicals produced by the strain S2 in PDB and sawdust media were determined using an TOFMS/GC–MS (Agilent, Santa Clara, CA, USA). A total of 52 and 21 odor chemicals were detected in the inoculated PDB and sawdust media, respectively. In the PDB medium, the top three odor chemicals comprised 4-hepten-3-one, 2-PE, and azulene, with the percentages of 14.7%, 12.6%, and 4.5%, respectively (Fig. 1A). In the sawdust medium, the top three odor chemicals were piperonal, benzaldehyde, and 2-PE, with the percentage of 28.2%, 11.6%, and 4.7%, respectively. 2-PE was undetected in the uninoculated PDB and sawdust media. These results indicated that *A. stygium* produces large amounts of 2-PE in different media.

### Best producer strain isolation and identification

**Fig. 2:**
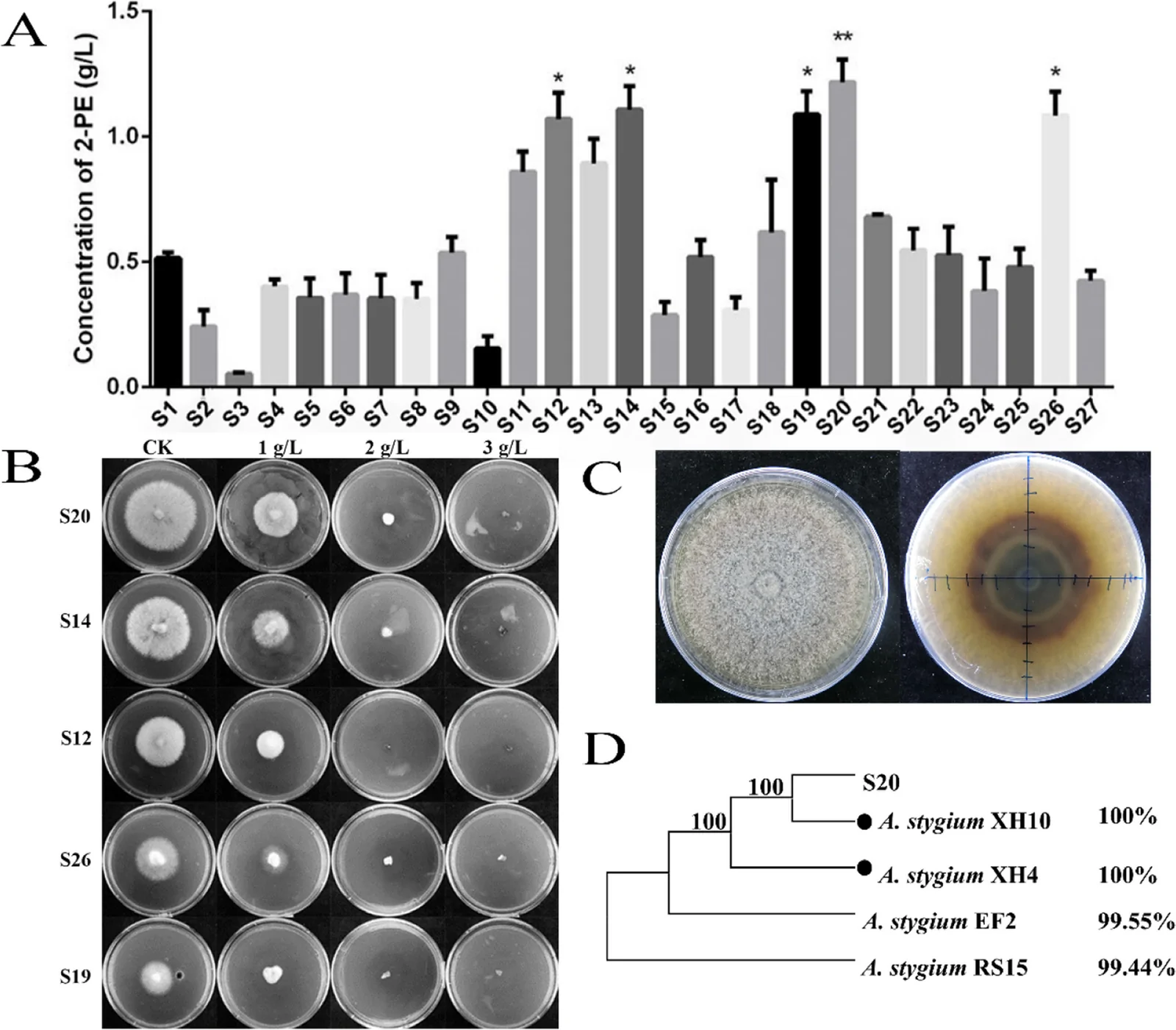
High-yield 2-PE strain screening and identification of *A. stygium*. **A** High-yield 2-PE strain screening after cultivated in PDB medium for 6 days at 28 °C. **B** Test for strain tolerance against 2-PE. CK, PDA medium without 2-PE addition. **C** Colonial morphology of strain S20. **D** Phylogenetic tree built by ITS sequences (ITS1-ITS4). Percentage on the right means sequence similarity of each strain compared with S20. *A. stygium* XH10 (accession number, FJ848859.1); *A. stygium* XH4 (accession number, FJ848853.1); *A. stygium* EF2 (accession number, MG881822.1); *A. stygium* RS15 (accession number, KF612313.1)

Next, a larger collection of *A. stygium* strains was screened in order to select the best 2-PE producer. After 6 days of culture at 28 °C in PDB medium, all the 27 tested *A. stygium* strains were found to produce 2-PE. Among them, five strains (strain S12, strain S14, strain S19, strain S20, and strain S26) produced 2-PE at a concentration exceeding 1.00 g/L, while the S20 strain produced up to 1.23 g/L of 2-PE (Fig. 2A, Table 2). After 4 days of culture at 28 °C, the concentration of 2-PE produced by the S20 strain in the PDB medium reached 38 mg/L; however, L-Phe was not detected. In contrast, the 2-PE concentration increased to 300 mg/L in the PDB + L-Phe medium, and the L-Phe concentration decreased from 4.0 to 1.5 g/L (Table 3).

**Table 3:** Concentration of 2-phenylethanol and L-phenylalanine produced by the S20 strain in different media

ID	PDB				PDB-L				Sig.^a^
	rp^b^1	rp2	rp3	rp4	rp1	rp2	rp3	rp4	
2-PE (mg/L)	39	36	38	38	333	269	348	250	**
L-Phe (g/L)	0	0	0	0	2.55	2.38	2.16	2.44	**

^a^Sig., significant difference at *p* < 0.05 (*) and* p* < 0.01(**) level

^b^rp, experimental replication

During the subsequent tolerance test among the five strains (strain S12, strain S14, strain S19, strain S20, and strain S26), the S20 strain had the highest mycelial growth rate in the PDA medium with the same concentration of 2-PE (Fig. 2B), indicating that S20 had the strongest tolerance against 2-PE. The mycelia of the S20 strain grew densely, emitted a rose smell, and secreted melanin after 7 days of cultivation, which resulted in the entire medium turning black (Fig. 2C). All these are typically morphological and odor characteristics of *A. stygium* (Deng et al. 2016). PCR with primers ITS1 and ITS4 of the S20 strain generated a single molecule of 873 bp. Sequence alignment and phylogenetic tree showed that the ITS sequence of the S20 strain had the highest similarity with that of *A. stygium*, showing 100% sequence identity with *A. stygium* XH4 (accession number, FJ848853.1) and *A. stygium* XH10 (accession number, FJ848859.1), respectively (Fig. 2D).

### Optimization of growth media

**Fig. 3:**
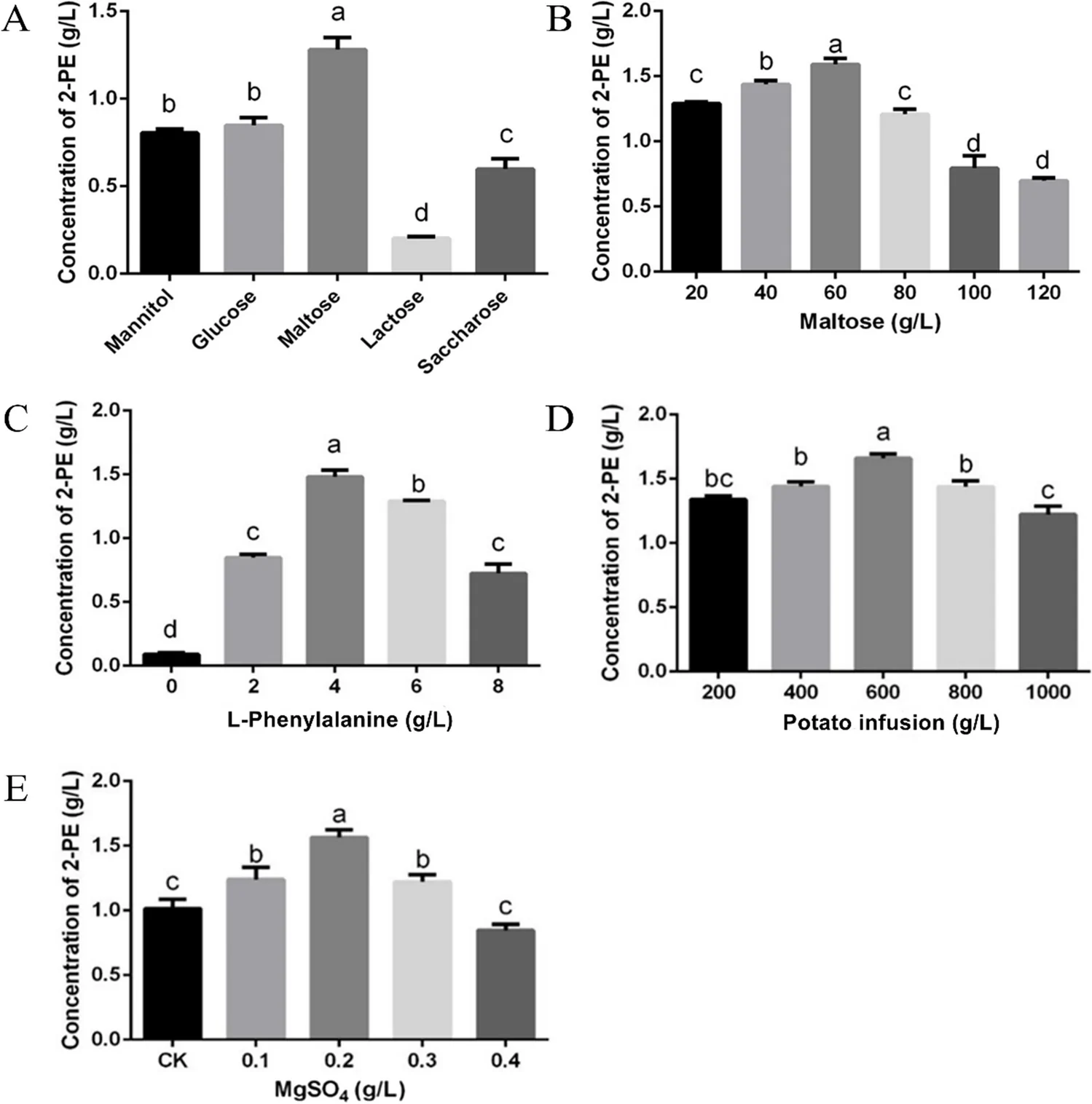
Optimization of growth media for maximum production of 2-PE by strain S20 cultivated 2 days at 28 ℃. **A**–**E** Effect of different carbon sources, maltose concentration, L-Phe supplemental level, potato dosage, and MgSO_4_ dosage, respectively

To evaluate the 2-PE synthesis capability of the S20 strain, optimal carbon sources were determined. As shown in Fig. 3A, the 2-PE concentration was the highest when maltose was used as the carbon source. When the initial maltose concentration was 60.00 g/L, the 2-PE concentration reached a maximum of 1.33 g/L after 48 h of cultivation (Fig. 3B).

As a nitrogen source, L-Phe plays a crucial role in 2-PE production (De Lima et al. 2018); therefore, it was necessary to explore the optimal addition of L-Phe to the medium. The 2-PE concentration peaked at an L-Phe concentration of 4.00 g/L but decreased at higher L-Phe concentrations (Fig. 3C). The increasing trend of 2-PE production with L-Phe concentration was consistent with the initial maltose concentration on 2-PE production; these results indicated that the concentration of biosynthesis 2- PE in *A. stygium* was lower with less additions of maltose and L-Phe in the medium.

Different potato infusion content affected 2-PE production. 2-PE production was higher when the potato infusion content was between 200.00 and 600.00 g/L but lower at a higher potato infusion (Fig. 3D). Previous studies have shown that Mg^2+^ enhanced the activity of dehydrogenase and decarboxylase (Hirano et al. 2007; Hirata et al. 2016). The effect of MgSO_4_ concentration on 2-PE production is shown in Fig. 2E. 2-PE production was observed to increase until the MgSO_4_ concentration reached 0.20 g/L.

Based on the single-factor experiments, crucial experimental parameters affecting 2-PE production were optimized, including potato infusion content and maltose, L-Phe, and MgSO_4_ concentrations (Table 2). According to the orthogonal test, it can be concluded that the primary and secondary factors affecting 2-PE production were L-Phe concentration and potato infusion. The optimal potato infusion, maltose, L-Phe, and MgSO_4_ concentration were set at 600.00 g/L, 40.00 g/L, 6.00 g/L, and 0.30 g/L, respectively (Table 4). The 2-PE concentration measured using this formula was 2.33 g/L.

**Table 4:** Data from the orthogonal growth and 2-PE production test performed with strain S20

Run	A	B	C	D	2-PE (g/L)
1	1	1	1	1	0.8101 ± 0.023
2	1	2	2	2	1.7798 ± 0.099
3	1	3	3	3	2.1201 ± 0.102
4	2	1	2	3	2.0141 ± 0.122
5	2	2	3	1	2.2312 ± 0.039
6	2	3	1	2	0.9012 ± 0.067
7	3	1	3	2	2.1726 ± 0.102
8	3	2	1	3	0.9772 ± 0.081
9 K_1_ K_2_ K_3_	3 4.7100 5.1465 5.0621	3 5.0004 4.9882 4.9336	2 2.6885 5.7062 6.5275	1 4.9536 4.8536 5.1114	1.9123 ± 0.178
k_1_	1.5700	1.6668	0.8962	1.6512	
k_2_	1.7155	1.6627	1.9021	1.6178	
k_3_	1.6873	1.6445	2.1758	1.7038	
R	0.1455	0.0223	3.839	0.086	
Order	R_C_ > R_A_ > R_D_ > R_B_				
Optimal combination	A_2_B_1_C_3_D_3_				

### Optimization of culture conditions

**Fig. 4:**
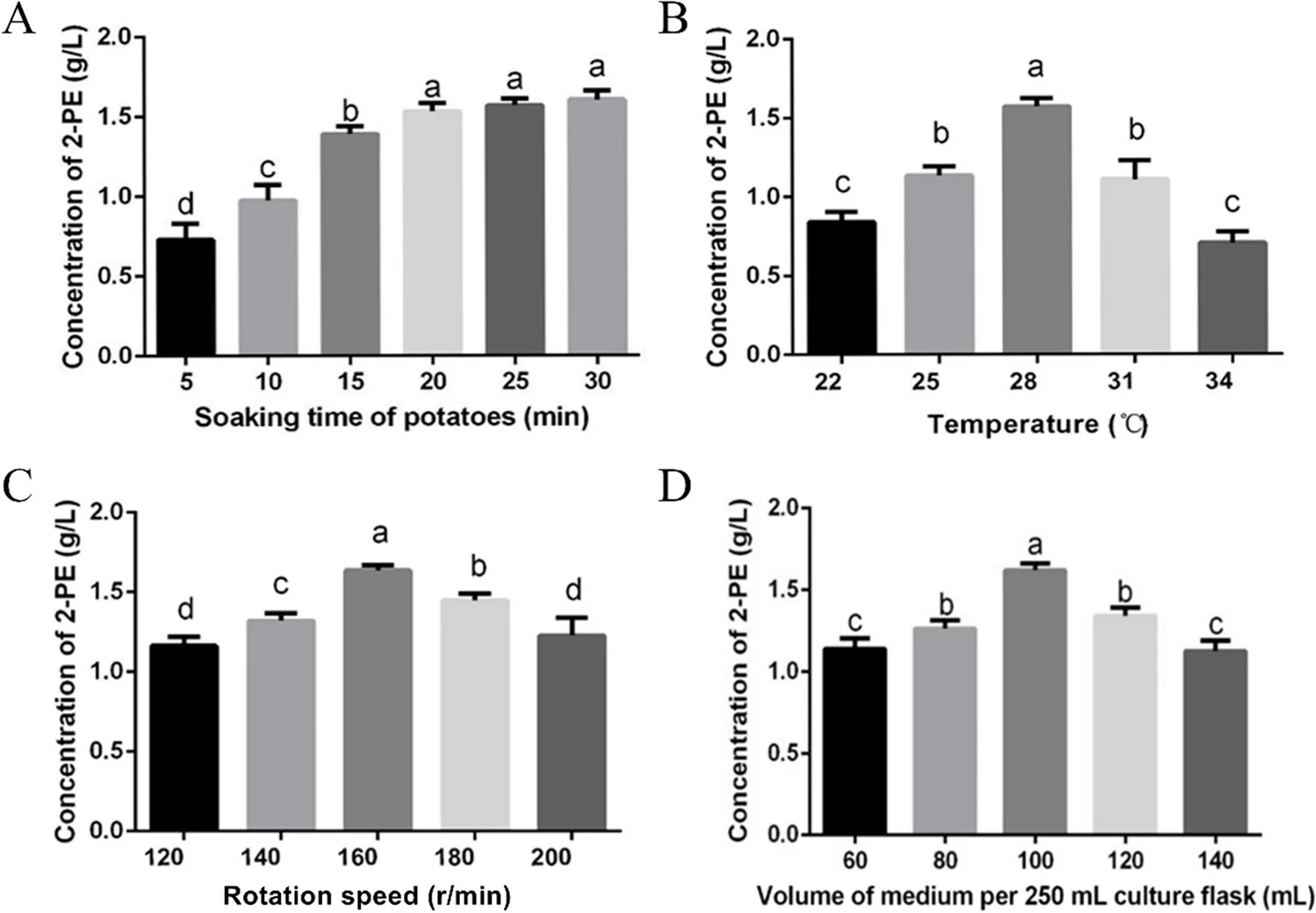
Optimization of culture conditions for maximum production of 2-PE by strain S20 cultivated for 2 days at 28 °C. **A**–**D** Effect of different potato extraction time, culture temperature, rotate speed, and liquid content, respectively

To improve the concentration of 2-PE, culture conditions were optimized based on the defined medium. As shown in Fig. 4A, the 2-PE concentration increased with increasing soaking time of potatoes. The 2-PE concentration was highest when the soaking time reached 30 min.

The optimization of temperature was evaluated every two centigrade from 22 to 34 °C (Fig. 4B). The 2-PE concentration increased as the temperature increased and then decreased at higher temperatures. The results showed that the 2-PE concentration highest value at 28 °C which is consistent with the optimal growth temperature range of *A. stygium* (Mu 2012).

Dissolved oxygen was also a factor that affected the 2-PE concentration of the S20 strain. The rotational speed and loading volume are shown in Fig. 4C and D, respectively. The results showed that the S20 strain inoculated into 100 mL PDB medium with vigorous agitation at 160 rpm was best.

### Analyses of the 2-PE synthesis pathway

To date, no studies have reported the ability of *A. stygium* to produce 2-PE. The genes involved in the production of 2-PE and metabolism of L-Phe in *S. cerevisiae* were retrieved. These genes were then compared with the complete genome sequence of *A. stygium* using Blast analysis (https://blast.ncbi.nlm.nih.gov/Blast.cgi), which could help speculate on the biosynthesis pathway of 2-PE in *A. stygium*.

In *S. cerevisiae*, nine genes (*ARO3*, *ARO1*, *ARO2*, *ARO7*, *PHA2*, *ARO8*, *ARO10*, *ADH*, and *PDR12*) and the Ssy1p membrane protein (encoded by the *SSY1* gene) have been identified to be involved in 2-PE synthesis (Delaney et al. 2023; Hazelwood et al. 2008; Larroude et al. 2021; Zhu et al. 2021). The first five genes (*ARO3*, *ARO1*, *ARO2*, *ARO7*, and *PHA2*) play important roles in the pathway and are primarily responsible for the conversion of glucose to phenylpyruvate. Phenylpyruvate is then converted to 2-PE via a series of reactions. Each of these genes had at least one homologue in the S20 strain genome. *ARO3* and *ARO1* had two homologues each in the S20 strain. *ARO2*, *ARO7*, and *PHA2* genes each had one homologue in the S20 strain genome (Table 5). The last four genes (*ARO8*, *ARO10*, *ADH*, and *PDR12*) encode relevant enzymes in the Ehrlich pathway that catalyze the conversion of L-Phe to 2-PE. The *SSY1* and *PDR12* genes had nine and six homologues in the S20 strain genome (accession number, JBAIZS000000000), respectively. *ARO8*, *ARO10*, and *ADH* had four, two, and seven homologues in the S20 strain genome, respectively.

**Table 5:** Genes and gene expression levels corresponding to the pathway of biosynthesis of 2-PE in *A. stygium*

Yeast gene	*A. stygium* gene	PDB			PDB + L-Phe^a^			Log2FC	Sig.^b^
		rp^c^1	rp2	rp3	rp1	rp2	rp3		
*ARO3*	*TJAS01_V10086660*	18.5	26.4	18.3	5.3	5.7	5.5	-1.94	**
	*TJAS01_V10043640*	0	0	0	0	0	0	0	-
*ARO1*	*TJAS01_V10004040*	5.2	6.4	4.9	1.1	3.6	1.6	− 1.4	*
	*TJAS01_V10066810*	3.53	7.49	5.41	1.16	0.53	0.61	− 1.94	**
*ARO2*	*TJAS01_V10058490*	32.3	37.6	41.9	12.8	14.6	10.8	− 1.55	*
*ARO7*	*TJAS01_V10037870*	19.7	28.1	37	10.1	6.1	8.9	− 1.74	*
*PHA2*	*TJAS01_V10028710*	5.5	6.91	11.2	2.35	0.66	1.51	− 2.39	**
*SSY1*	*TJAS01_V10057390*	5.63	7.95	10.37	13.97	12.77	12.53	0.71	*
	*TJAS01_V10011140*	0	0	0	0	0	0	0	-
	*TJAS01_V10044110*	10.99	17.1	12.4	30.3	28.99	35.65	1.64	**
	*TJAS01_V10031850*	0	0	0.52	0	0.28	0	− 0.86	-
	*TJAS01_V10073660*	9.47	9.61	9.44	38.3	40.02	79.29	2.47	**
	*TJAS01_V10064940*	0	0	0	0	0	0	0	-
	*TJAS01_V10054420*	4.56	6.45	6.14	3.81	1.91	3.96	− 0.84	-
	*TJAS01_V10088760*	2.58	3.06	4.36	1.81	0.64	0	2.03	**
	*TJAS01_V10068300*	0.88	0.57	0.9	1.01	0.9	0.31	− 0.07	-
*ARO8*	*TJAS01_V10003540*	19.8	12	11.6	27.1	27.3	65.7	1.48	**
	*TJAS01_V10041730*	56.5	63.4	66.3	95.5	88.9	101.1	0.61	*
	*TJAS01_V10050660*	11.9	3.4	14.6	285	343	76.7	4.6	**
	*TJAS01_V10058090*	47.72	47.31	60.92	100.6	89.1	79.98	0.79	*
*ARO10*	*TJAS01_V10069960*	320.2	402.5	301.8	435	402	689.8	0.58	*
	*TJAS01_V10009870*	2.8	5.1	15.2	24.9	26.9	30.6	1.65	**
*ADH*	*TJAS01_V10028140*	617.4	576.5	401.2	939	810	992.1	0.78	*
	*TJAS01_V10101530*	1.58	0.86	0.49	1.11	0.58	0	− 0.81	-
	*TJAS01_V10067110*	6.28	6.35	4.54	3.89	1.01	4.54	− 0.86	-
	*TJAS01_V10064980*	0	0.29	0.65	0.52	0.15	0.68	0.54	-
	*TJAS01_V10081920*	0.88	0.97	3.16	19.68	14.71	38.28	3.86	*
	*TJAS01_V10098460*	7.28	9.93	11.88	15.56	11.57	20.51	0.7	-
	*TJAS01_V10101010*	0.6	0.4	0.8	1.5	1.8	2.2	1.61	**
*PDR12*	*TJAS01_V10086000*	23.1	33.45	19.3	100.1	78.9	89.06	1.82	**
	*TJAS01_V10054500*	1.18	3.76	1.77	10.24	8.22	23.1	2.63	**
	*TJAS01_V10004470*	0.77	3.56	0.61	5.26	4.49	4.45	1.55	*
	*TJAS01_V10066070*	3.16	6.66	3.09	9.98	12.68	5.51	1.12	*
	*TJAS01_V10090430*	0.47	0.73	1.05	0.3	0.67	0	− 1.21	*
	*TJAS01_V10055750*	0	0	0	0	0	0	0	-

Log2FC − PDB + L-Phe divided by PDB divided by logarithm base 2

*Corrected *p*-value < 0.05 level was significantly different

**Corrected *p*-value < 0.01 level was significantly different

^a^PDB + L-Phe − PDB + 4 g/L L-phenylalanine

^b^Sig., significance difference

^c^rp, experimental replication

**Fig. 5:**
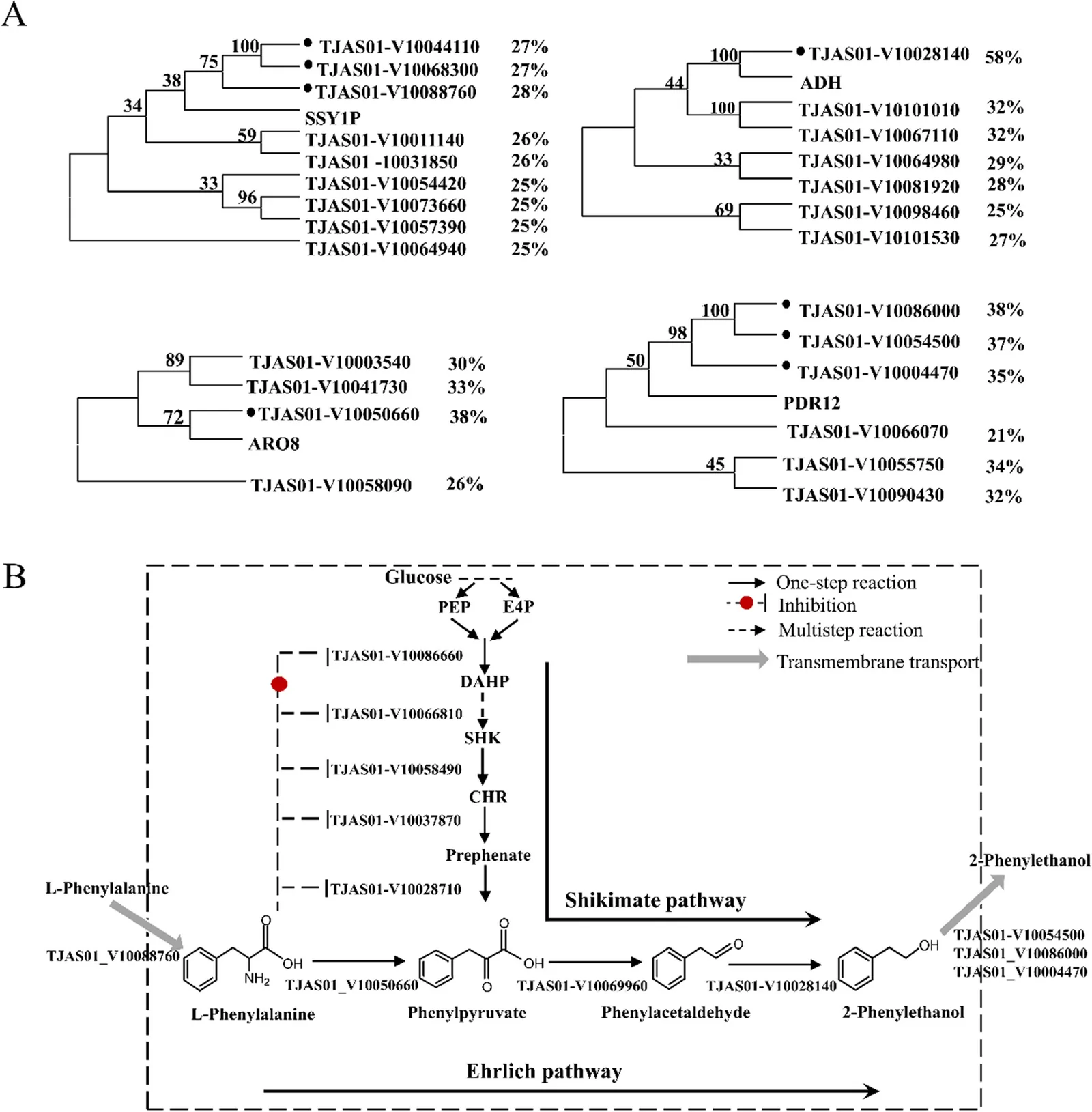
Prediction of key genes and pathway for biosynthesis 2-PE in *A. stygium*. **A** Phylogenetic trees constructed by protein sequences corresponding to Ssy1p (accession number, NP_010444.1; upper left), ADH (accession number, NP_014032.1; upper right), ARO8 (accession number, NP_011313.1; low left), and PDR12 (accession number, NP_015267.1; low right) of *S. cerevisiae*. Percentage on the right means protein similarity of each gene compared with *A. stygium* homologues. **B** Possible pathway of biosynthesis 2-PE in *A. stygium*. PEP, phosphoenolpyruvate; E4P, erythrose-4-phosphate; DAHP, 3-deoxy-D-arabinoheptulosonate; SHK, shikimate; CHR, chorismic acid

Ssy1p is a membrane protein with a function to import L-Phe from the extracellular environment (Delaney et al. 2023). It is encoded by the member of the family of amino acid transporter genes, *SSY1* (Didion et al. 1998). Nine genes in the S20 strain genome were detected by doing tBLASTx searches the *S. cerevisiae* genome. According to the phylogenetic tree, the products of the genes *TJAS01-V10044110*, *TJAS01-V10088760*, and *TJAS01-V10068300* had a close genetic relationship with Ssy1p of *S. cerevisiae* (Fig. 5A). When 4 g/L of L-Phe was added to the PDB medium, the gene expression level of *TJAS01-V10088760* was significantly upregulated, whereas those of *TJAS01-V10044110* and *TJAS01-V10068300* were significantly downregulated or remained unchanged, respectively (Table 5). Changes in gene expression levels of *TJAS01-V10088760* were compared to the gene function of *SSY1.* As a result, *TJAS01-V10088760* was most likely to be a *SSY1* candidate in the S20 strain. *TJAS01-V10005660*, *TJAS01-V10069960*, *TJAS01-V10028140*, and *TJAS01-V10054500* were most likely to be *ARO8*, *ARO10*, *ADH*, and *PDR12*, respectively, in the Ehrlich pathway (Fig. 5B). In addition, upon the addition of L-phenylalanine to the medium, the expression levels of genes in the Ehrlich pathway were higher than those in the shikimate pathway, suggesting that the synthesis of 2-PE through the Ehrlich pathway was higher than that through the shikimate pathway in the S20 strain (Fig. 5B). The 2-PE synthesis pathway in the S20 strain was thus similar to that in *S. cerevisiae* (Dai et al. 2021; Hazelwood et al. 2008).

## Discussion

Currently, studies on the biosynthesis of 2-PE have focused primarily on yeasts and few on filamentous fungi, such as some species of *Aspergillus* (Vázquez et al. 2022). Based on GC–MS analysis, Wani et al. (2010) found that *Aspergillus niger* JUBT 3 M was able to produce 2-PE at a lower concentration than many of the tested yeasts. After optimizing the culture conditions, Etschmann et al. (2015) used *A. niger* DSM 821 to produce 2-PE at concentrations as high as 1.43 g/L. Under the optimal glucose and L-Phe concentration, the 2-PE concentration of *A. oryzae* RIB40 may reach up to 0.1 g/L (Masuo et al. 2015). In the present study, 2-PE was detected as one of the main components emitted from the *A. stygium* mycelia grown in bothPDB and sawdust media. Among 27 *A. stygium* isolates screened in this study, the strain “Jinjiling” (strain S20) showed the highest production of 2-PE and had the strongest tolerance of the product. By optimizing the initial concentrations of maltose, L-Phe, potato infusion, and MgSO_4_, the concentration of 2-PE could be as high as 2.33 g/L, which is a value relatively higher than those among other filamentous fungi reported (Etschmann et al. 2015; Masuo et al. 2015). As a result, *A. stygium* has great potential as a filamentous candidate for the high production of 2-PE.

In addition to *S. cerevisiae* (Dai et al. 2021; Zhu et al. 2021), many other yeast species, such as *P. fermentans* (Fan et al. 2020; Mierzejewska et al. 2019), *K.marxianus* (De Lima et al. 2018; Etschmann and Schrader 2006; Li et al. 2021), *Yarrowia lipolytica* (Gu et al. 2020a, b), *Zygosaccharomyces rouxii* (Dai et al. 2020), and *Metschnikowia pulcherrima* (Chantasuban et al. 2018; Zhu et al. 2022), have been reported to synthesize 2-PE from glucose through the shikimate pathway and from L-Phe through the Ehrlich pathway.

The production of 2-PE through the Ehrlich pathway in *Aspergillus* sp. is not as widely studied as in yeasts (Etschmann et al. 2015). *A. niger* CMICC 298302 was firstly reported in filamentous fungi to produce 2-PE using L-Phe as the nitrogen source (Lomascolo et al. 2001). *Ashbya gossypii* is a filamentous ascomycete that harbors genes for aromatic amino acid catabolism (*ARO8a*, *ARO8b*, *ARO10*, and *ARO80*) and produces high concentrations of 2-PE. Deletion of these genes, except for *AgARO8a*, strongly impairs the production of 2-PE, indicating that the Ehrlich pathway plays an important role in 2-PE production (Ravasio et al. 2014). Both Ehrlich and PEA (phenylethyl alcohol) pathways were responsible for the synthesis of 2-PE in *A. oryzae* RIB40. In the PEA pathway, phenylalanine was decarboxylated to phenylethylamine, which was then oxidatively deaminated to form phenylacetaldehyde, and subsequently dehydrogenated to yield 2-PE (Tieman et al. 2006). However, when L-Phe was added in the culture medium, the isolate synthesized 2-PE primarily via the Ehrlich pathway (Masuo et al. 2015). Genome comparisons revealed that each key gene in the *S. cerevisiae* shikimate and Ehrlich pathways had one or more homologous genes in *A. stygium*. When L-Phe was added to the PDB medium, the amount of 2-PE produced by *A. stygium* increased rapidly, which was consistent with that produced by *S. cerevisiae* (Dai et al. 2021)*.* Gene expression levels of candidates in the Ehrlich pathway were upregulated when L-Phe was added to the medium. Based on the above evidence, it is speculated that *A. stygium* has a synthetic pathway similar to that of *S. cerevisiae* for the production of 2-PE. More evidence, such as gene knockout, is essential to further confirm the shikimate and Ehrlich pathways and their corresponding genes in *A. stygium.*

Each year, agro-food industries produce large quantities of residues, and their utilization as a cheap substrate for the production of 2-PE has many advantages, such as abundant substrate resources, reduced cost, and environmental friendliness (Mitri et al. 2022). Many studies have tested various agro-industrial waste and by-products for raw materials to produce 2-PE, including whey (Chreptowicz et al. 2018), grape must (Garavaglia et al. 2007), corn stover (Mierzejewska et al. 2019), sugar beet molasses (Martínez-Avila et al. 2018; Martínez et al. 2018b; Zhan et al. 2020), sugarcane bagasse (Martínez-Avila et al. 2018, 2020; Martínez et al. 2018a), tobacco (Wang et al. 2013), and cassava wastewater (Oliveira et al. 2015). Most spent compost of *T. fuciformis* substrate contains only *A. stygium* and no other microorganisms (Liu et al. 2019) and is considered an ideal material for direct extraction of 2-PE. *T. fuciformis* has been cultivated in Northeast Asia since the 1960s (Sun 2023). In 2020, more than 500 kt of fresh *T. fuciformis* was produced (Sun 2023), generating more than 800 kt of substrate waste. The mycelia of *A. stygium* spread over the entire substrate and provide nutrition for *T. fuciformis* (Deng et al. 2018). However, the growth of *T. fuciformis* mycelia is limited to the region (20–30 mm in diameter) near the inoculation area (our unpublished observations). As a result, approximately 95% of *T. fuciformis* substrate contains only *A. stygium* and no other microorganisms and is considered an ideal material for direct extraction of 2-PE. Further studies are necessary to construct a cultivation system of *T. fuciformis*, including optical strains, culture medium, and cultivation conditions, to obtain high yield of *T. fuciformis* and good 2-PE production at the same time. Our study on high-efficiency 2-PE biosynthesis in *A. stygium* not only makes it possible to reuse the spent compost substrate of *T. fuciformis* cultivation but also provides an alternative option for the production of natural 2-PE.

In this study, 2-PE was detected in *A. stygium* mycelial growth medium. The strain “Jinjiling” (strain S20) produced the highest 2-PE concentration and had the strongest tolerance of the product. By optimizing the initial concentrations of maltose, L-Phe, potato infusion, and MgSO_4_, the concentration of 2-PE was as high as 2.33 g/L. The pathway for the biosynthesis of 2-PE in *A. stygium* was similar to that in *S. cerevisiae*. In summary, *A. stygium* has great potential to utilize substrate waste of *T. fuciformis* cultivation to produce high yields of 2-PE.

## Data Availability

The datasets generated during the study are available from the corresponding author on reasonable request. Illumina sequencing data for six RNA sequencing have been deposited in the NCBI with the accession number PRJNA1018449.
